# Development and validation of a questionnaire to assess the health related Social Capital for Chronic Kidney Disease among Mexican adolescents

**DOI:** 10.1371/journal.pone.0328386

**Published:** 2025-07-21

**Authors:** Carolina Quiñones-Villalobos, Carlos Alberto Prado-Aguilar, Gregorio Tomás Obrador-Vera, José Manuel Arreola-Guerra, Jannett Padilla-López, Alicia Alanis-Ocádiz, Diana Lorena Cisneros-García, Diana Cristina Navarro-Rodriguez, Laura Leticia Tirado-Gómez

**Affiliations:** 1 Programa de Doctorado en Ciencias Médicas, Odontológicas y de la Salud, Universidad Nacional Autónoma de México, Mexico City, Mexico; 2 Coordinación Auxiliar de Investigación en Salud, Instituto Mexicano del Seguro Social, Aguascalientes, Aguascalientes, Mexico; 3 Departamento de Epidemiología y Salud Pública, Escuela de Medicina de la Universidad Panamericana, Mexico City, Mexico; 4 Departamento de Nefrología, Centenario Hospital Miguel Hidalgo, Aguascalientes, Aguascalientes, Mexico; 5 Unidad de Medicina Familiar, Instituto Mexicano del Seguro Social, Aguascalientes, Aguascalientes, Mexico; 6 Departamento de Salud Pública, Facultad de Medicina, Universidad Nacional Autónoma de México, Mexico City, Mexico; University of Helsinki, FINLAND

## Abstract

**Background:**

Social Capital on health has been studied widely, to date there is no valid and reliable questionnaire that measure it in Chronic Kidney Disease (CKD).

**Objective:**

To develop, validate and assess the reliability of Social Capital related to CKD questionnaire for Mexican adolescents.

**Methods:**

An instrumental study was employed to validate a questionnaire that assesses the cognitive and structural domains of Social Capital related to CKD. The questionnaire was generated by operationalization of the constructs and validated by assessing the content, face validity, criteria and construct validity. Reliability was assessed through the Cronbach´s alpha.

**Results:**

The content validity of the questionnaire was confirmed through Kendall’s W of 0.925 (p = 0.01) and its face validity was evaluated by four focus groups. A principal component analysis on a sample of 281 adolescents indicated that 72.78% of the variance was explained by the cognitive domain and 83.20% by the structural domain. A confirmatory analysis returned a chi-squared value of 142.99 (p = 0.05), a CFI of 0.97, a TLI of 0.96, a RMSEA of 0.040 and a SRMR of 0.07 for the cognitive domain. Similarly, a chi-squared of 408.296 (p < 0.001), a CFI of 0.98, a TLI of 0.97, a RMSEA 0.03 and a SRMR of 0.06 were returned for the structural domain. The validity of the criteria was assessed through a Pearson’s correlation for both the cognitive and structural domains. There was a mild-to-strongly significant correlation (p ≤ 0.001) among items and dimensions within each domain, with correlation coefficients ranging from 0.23 to 0.83. As a determinant of the reliability of the questionnaire, the Cronbach’s alpha was 0.84 and 0.94 for the cognitive and structural domain, respectively.

**Conclusions:**

A valid and reliable questionnaire has been developed to measure the influence of Social Capital on health in relation to CKD among Mexican adolescents.

## Introduction

There is evidence that Social Capital (SC) affects a variety of health problems, such that a high level of SC has been associated with better health, social, political and economic outcomes [1]. Indeed, SC has been defined by Putnam as “*features* of *social organization like networks*, *norms* and social *trust* that *facilitate coordination* and cooperation for *mutual benefit”* [1]. One of the most widely accepted approaches to study SC involves its measurement through two domains: structural (SD) and cognitive (CD) [2]. Both these domains are interrelated, and their origin resides in an individual’s mental and affective processes. The SD can be measured objectively since it refers to social processes and aspects of social organization. By contrast, CD is measured subjectively, and it refers to what people think and perceive about their social relationships. Both these domains foster productivity and security in individuals, facilitating cooperation and collective action, and influencing the way people relate to each other [2]. Each of the two domains of SC can be considered to be comprised of multiple dimensions depending on the object of the study and the context [3]. For example, the mechanisms by which SC can help improve an individual’s health have been proposed to involve collective socialization, informal social control and collective efficacy [3–6].

Chronic Kidney Disease (CKD) is defined as the presence of structural or functional abnormalities of the kidney that persist for more than 3 months. CKD is defined by indicators of kidney damage and/or a glomerular filtration rate of ≤60 mL/min/1.73m^2^ [7]. It is a disease with a significant impact on an individual´s health and a strong economic impact. Moreover, it currently affects more than 850 million people worldwide, with an estimated 14.5 million people having end-stage CKD by 2030 [8,9]. In Aguascalientes (Mexico), the prevalence of CKD has been increasing in recent years, in 2020 it was 2075 people per million population (pmp), and in 2022 it was 2328 pmp, ranking seventh worldwide behind Taiwan, Korea, Japan, Thailand, USA and Singapore [10–12]. This prevalence has a bimodal distribution, with a peak among individuals aged 20–40 years old where the highest frequency is found, and a second peak among those aged 50–70 years old. In the 20–40 year old group, Aguascalientes has the highest prevalence worldwide and in this age group, the main cause of CKD in Aguascalientes is considered to be of “unknown cause” (73%) [13]. Hence, and in the absence of precipitating factors, screening studies have been carried out in adolescents in this state that identified a CKD incidence (Stage I G1A2) of 3.7% (95% Confidence Interval 2.1–5.3) [14].

CKD has yet to be approached from a primarily clinical perspective, evaluating the risk factors associated with the progression and treatment of this disease [15]. In this sense, it is important to note the benefits that can be gained by studying the impact of SC on chronic degenerative diseases and in vulnerable populations like adolescents, in line with the social determinants of health model [16]. SC is considered an intermediate determinant, these determinants are established through social, economic and political mechanisms give rise to a set of socioeconomic positions whit are stratified and determine differences in exposure and vulnerability to health-compromising conditions. This approach implies that decisions that groups or individuals make, in relation to their lifestyle and behavioral habits, cannot be considered outside the social context where such choices take place [16].

The influence of SC has yet to be evaluated in relation to CKD. Indeed, while multiple questionnaires have been developed and validated to measure SC, there is still no consensus on the theoretical approaches to be used or on the specific dimensions to be studied. Indeed, the questionnaires developed to date often present methodological weaknesses in terms of their validity and reliability. In a literature search to identify studies of SC related to health that were carried out on adolescents, 40 studies were identified that addressed mental health [17–30], addiction [31–39], general health [40–47], reproductive health [48–50] and violence [51–53]. In seven of these studies, the theoretical approach used was reported but not described [27–29,45,46,49,53] and in 14, no theoretical approach was included [17–19,21,33–35,40,42,48,51,52,54,55]. Specific domains were identified in some studies as dimensions to address community or structural SC [19,21,22,25,30,35,37,43,51,54], and cognitive or individual SC domains [19,21,37,43,51]. As such, trust was a dimension studied in 35% of the 40 studies identified [20,26,28,31,35,37–39,43,45,46,49–51], followed by participation in 17.5% [28,31,35,37,43,46,49], generalized norms in 15% [20,28,35,38,39,50], groups and networks in 12.5% [38,39,46,50,51], horizontal SC in 12.5% [27–29,43,56], family cohesion in 10% [18,28,38,44], and social support [20,30,46] and bridging social capital [29,43,56] each in 7.5% of the studies identified. Four studies identified two dimensions (family interactions [18,53] and volunteering [48,49]), whereas seven studies identified 1 dimension (charitable donations [48], political awareness [48], collective action [51], cooperation [51], family control [18], family support [52], and feeling of belonging [45]), whereas no dimension was reported in ten studies [17,22,32–34,36,40–42,47].

Although SC has been studied in relation to different health problems in adolescent populations, none of them reports the use of a questionnaire that meets all the criteria for development, validation, and reliability. Based on the above, the aim of the present study was to develop a questionnaire to measure the SC related to CKD among Mexican adolescents, and to evaluate its validity and reliability.

## Materials and methods

Cross-sectional study of a SC questionnaire focused on CKD was carried out on adolescents in the state of Aguascalientes. The participants were recruited from a Project carried out to detect CKD among adolescents in this state. In a random sample, 281 adolescents aged 14 or 15 years of age were selected, without any information regarding their kidney function. These were third year students recruited from a random sample of 47 secondary schools in the municipalities of Aguascalientes, Calvillo and Rincón de Romos.

### Design of the questionnaire

The questionnaire was constructed by operationalizing the SC construct based on an extensive literature review focusing on CKD. Initially 98 items were generated, representing the CD with 6 dimensions (generalized norms, social harmony, sense of belonging, perceived fairness, support and trust) and the SD through 9 dimensions (organizational participation, institutional links, frequency of action, network size, collective action, degree of citizenship, diversity, links to groups with resources, and links with parallel groups). The items were scored on a 5-point Likert scale and/or on a continuous scale (see the operationalization annex). In some of the questionnaire’s dimensions the student will be informed that the following questions are set-out based on the assumption that they are part of a neighborhood group or a committee of classmates with CKD.

### Validation of the questionnaire

The questionnaire was validated in four stages: content validation, face validity, construct validation, and criterion validation. The content validity was assessed by a panel of five experts through a mixed qualitative and quantitative approach based on the Delphi technique [57]. The criteria employed to select the experts were: having knowledge and experience in the methodology to design and validate measurement instruments; having experience in making evidence-based judgments and decisions; having an understanding of the social determinants of health and/or CKD; availability and motivation to participate; impartiality and other inherent qualities (trustworthy, adaptable, etc.) (60). In the qualitative stage, the experts reviewed the congruence of each item, ensuring that each corresponded to the concept and belonged to the appropriate dimension in accordance with the theory of SC [58–60]. In the quantitative stage, expert consensus was reached through the use of a form for expert judgment on the basis of four criteria (sufficiency, clarity, coherence and relevance) and analyzed using the Kendall’s W test [61].

Face validity was assessed through focus groups that set-out to analyze the elements of each item in the SC questionnaire, reaching a consensus and changing those elements that were not understood to ensure clarity [61]. This process was carried out in three steps, the first involving the formation of four groups, each made up of 8–12 adolescents enrolled at secondary schools. Subsequently, guidelines were prepared in sessions that lasted no longer than two hours. In these sessions, the moderator provided a brief introduction to the items in the SC questionnaire, and the sessions were organized to follow three phases: presentation, motivation dynamics, completion of the session and its closure. Each session was recorded and to be analyzed with the ATLAS.ti software (version 6.0). Finally, the third phase involved analyzing the sessions. This procedure commenced by defining a category tree based on the questionnaire’s guidelines, which was expanded by coding the text into four presumptive categories (technical concept, similarity concept, lack of clarity in the concept, or additional comments). Subsequently, one or more networks of the relationships between the technical concepts and their similarities were drawn up to generate flow diagrams of the interrelated categories. This allowed similar concepts to be identified so that the technical concept could be replaced or modified to clarify each of the items in the SC questionnaire [60].

The sample size for construct validity was determined as three adolescents per item, according to the earlier recommendations of the use of a graded scale of sample sizes when designing a questionnaire to achieve stability and replicability of the items [62]. Construct validation was carried out in two phases, involving an exploratory and a confirmatory analysis. The exploratory factor analysis was performed through a principal components analysis (PCA) with varimax rotation, which maximizes the sum of the variances of the squared loadings within each column of the matrix [63]. The assumption of normality was checked for each item using a skewness and kurtosis analysis (with values between −2 and +2 considered acceptable), the number of factors was defined based on operationalization, with an eigenvalue ≥0.4 established as the cut-off point for item inclusion, and Kaiser’s normalization was applied. Values for the Kaiser-Meyer-Olkin (KMO) test [64] of sampling adequacy and the Barlett test of Sphericity [65] were determined. The confirmatory factor analysis (CFA) was performed using structural equation modeling. The goodness-of-fit indices met the following cut-off criterion: root mean square error of approximation (RMSEA) ≤0.05, comparative fit index (CFI) ≥0.95, a Tucke-Lewis index (TLI) ≥0.95 and a standardized root mean square residual (SRMR) ≤0.08 [66].

The validity criterion was assessed through the Pearson´s correlation coefficients (PCCs) between the dimensions of each domain, and between the items and dimensions. The cut-off criteria were correlation coefficients values ≥0.5 and p value ≤0.05 for each domain of the questionnaire (CD and SD). Subsequently, the reliability of the questionnaire was examined through the Cronbach’s alpha value (αC) for all items in the questionnaire. The minimum criterion for acceptable reliability was αC ≥ 0.70 [58–60].

### Ethical considerations

The research protocol was approved by the Local Clinical Research Committee and the Ethics Committee at the General Hospital of Zone No. 1 of the Mexican Institute of Social Security in Aguascalientes (Registration No.: R-2021-101-06) and the Research Ethics Committee of the Centenario Hospital Miguel Hidalgo in Aguascalientes (Registration No.: 2022-U-04). Written approval to participate was obtained from the adolescents and written informed consent was obtained from their parents and/or guardians.

## Results

This study was carried out on 281 adolescents, 52.3% of whom were male and their mean age was 14.21 ± 0.48 (standard deviation). The participants responded to a questionnaire that was designed specifically to measure the SC related to CKD in adolescents. A mixed qualitative and quantitative approach was adopted to assess the validity of the questionnaire’s content. The qualitative assessment was carried out over four rounds, in which the experts reached a consensus after reviewing the theoretical congruence of the domains, dimensions and items in the questionnaire, having made changes to four items in the SD of the SC questionnaire. In the quantitative stage, expert agreement was demonstrated by achieving a Kendall’s W value of 0.925 (p = 0.011) in the fourth round, an indication of the strong agreement between the experts. Nevertheless, five items were removed due to a lack of theoretical congruence and the remaining 93 items were assessed in the subsequent validation stages.

Face validity was assessed by analyzing the discussions of the four focus groups to identify the technical concepts underlying the items and the similarities in the concepts proposed by the adolescents. As a result, 11 technical and similar concepts to the ones proposed by adolescents were identified in the CD and 13 in the SD. Modifications to clarify these items were introduced and accepted by experts.

To assess the construct validity, the assumption of normality in the items and dimensions was first verified using a skewness and kurtosis analysis on the CD and SD items, with values between −2 and +2 considered acceptable. The final model of the questionnaire had a parametric distribution, with the exception of two dimensions: trust and organizational participation. The mean and standard deviation ranged from 2.79 (± 0.78) to 4.49 (± 1.65) for the responses to the CD items and from 2.13 (± 0.89) to 4.14 (± 1.56) for the responses to the SD items.

The KMO test values obtained for the CD and the SD were 0.80 and 0.94, respectively. Bartlett’s Test of Sphericity returned a Chi-squared value of 3378.08 for the CD (with df = 820 and p = 0.001) and of 15,321.08 for the SD (with df = 1486 and p = 0.001), confirming the adequacy of the sample for the PCA. In the initial model of the PCA, the communality of the items ranged from 0.63 to 0.81 in the CD (Table 1) and from 0.82 to 0.97 in the SD (Table 2). All the dimensions in both domains had eigenvalues ≥ 1. A final model from the PCA was obtained for both domains and from the 93 items, 48 were eliminated in order to keep a parsimonious model with 3 items in each dimension. All the items that remained had factor loading ≥ 0.4 and accordingly, the CD with 6 dimensions and 18 items explained 72.78% of the variance (Table 1), while the SD with 9 dimensions and 27 items explained 83.20% of the variance (Table 2). The total variance explained was ≥ 70%, meeting the psychometric criteria (57).

**Table 1:** Final Principal Component Analysis by Cognitive Domain in the Social Capital Related to CKD questionnaire in Mexican Adolescents.

Items	Factor 1	Factor 2	Factor 3	Factor 4	Factor 5	Factor 6	Communality
GN2	0.5890						0.8127
GN7	0.5428						0.7573
GN8	0.5659						0.7822
SH5				0.5758			0.7219
SH6				0.4872			0.6993
SH7				0.6315			0.6820
SB3		0.5661					0.7799
SB4		0.5414					0.7953
SB5		0.5416					0.7726
PF2					0.5953		0.7063
PF4					0.5412		0.7486
PF5					0.5652		0.7750
S4			0.5574				0.7537
S5			0.5584				0.7656
S6			0.5897				0.7815
T4						0.5081	0.7473
T6						0.6019	0.6332
T7						0.6026	0.6365
% explained by factor variance	14.75	13.90	12.55	11.11	10.24	10.23	
% explained of the total scale variance	72.78						

GN: Generalized Norms; Factor 1. SH: Social Harmony; Factor 4, SB: Sense of Belonging; Factor 2. PF: Perceived Fairness; Factor 5. S: Support; Factor 3. T: Trust; Factor 6. KMO: Kaiser-Meyer-Olkin CD 0.80. Barlett. Test CD chi square 3378.08, 820 df, p = 0.001.

**Table 2:** Final Principal Component Analysis by Structural Domain in the Social Capital Related to CKD questionnaire in Mexican Adolescents.

Items	Factor 1	Factor 2	Factor 3	Factor 4	Factor 5	Factor 6	Factor 7	Factor 8	Factor 9	Communality
PO4									0.5563	0.8383
PO5									0.6236	0.8224
PO6									0.5373	0.8202
IL4b		0.5821								0.9197
IL5b		0.5602								0.8888
IL6b		0.5780								0.9194
FA1b				0.6042						0.9558
FA2b				0.5313						0.9258
FA5b				0.4531						0.9653
NS2b	0.4524									0.9573
NS4b	0.6444									0.9481
NS5b	0.4878									0.9588
CA2b								0.5451		0.9329
CA4b								0.5105		0.9331
CA5b								0.3843		0.9675
DC1			0.5733							0.9522
DC5			0.5723							0.9037
DC6			0.5364							0.9082
D1							0.5430			0.8801
D2							0.5667			0.8651
D4							0.5901			0.9066
LGR2					0.5881					0.8971
LGR3					0.5953					0.9058
LGR4					0.5207					0.9293
LPG4						0.5940				0.8853
LPG5						0.5778				0.8783
LPG6						0.5466				0.8781
% explained by factor variance	10.24	10.17	9.87	9.81	8.96	8.91	8.68	8.41	8.15	
% explained of the total scale variance	83.20									

PO: Organizational participation; Factor 9. IL: Institutional Links; Factor 2. FA: Frequency of Actions; Factor 4. NS: Network Size; Factor 1. CA: Collective Action; Factor 8. DC: Degree of Citizenship; Factor 3. D: Diversity, Factor 7. LGR: Links to Groups with Resources; Factor 5. LPG: Links with Parallel Groups; Factor 6. KMO: Kaiser-Meyer-Olkin SD:0.94. Barlett Test SD chi square 15,321.08, 1486 df p = 0.001.

**Fig 1:**
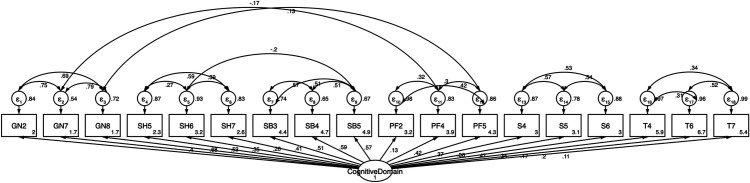
Confirmatory model by cognitive domain of the Social Capital Related to CKD questionnaire in Mexican Adolescents.

**Fig 2:**
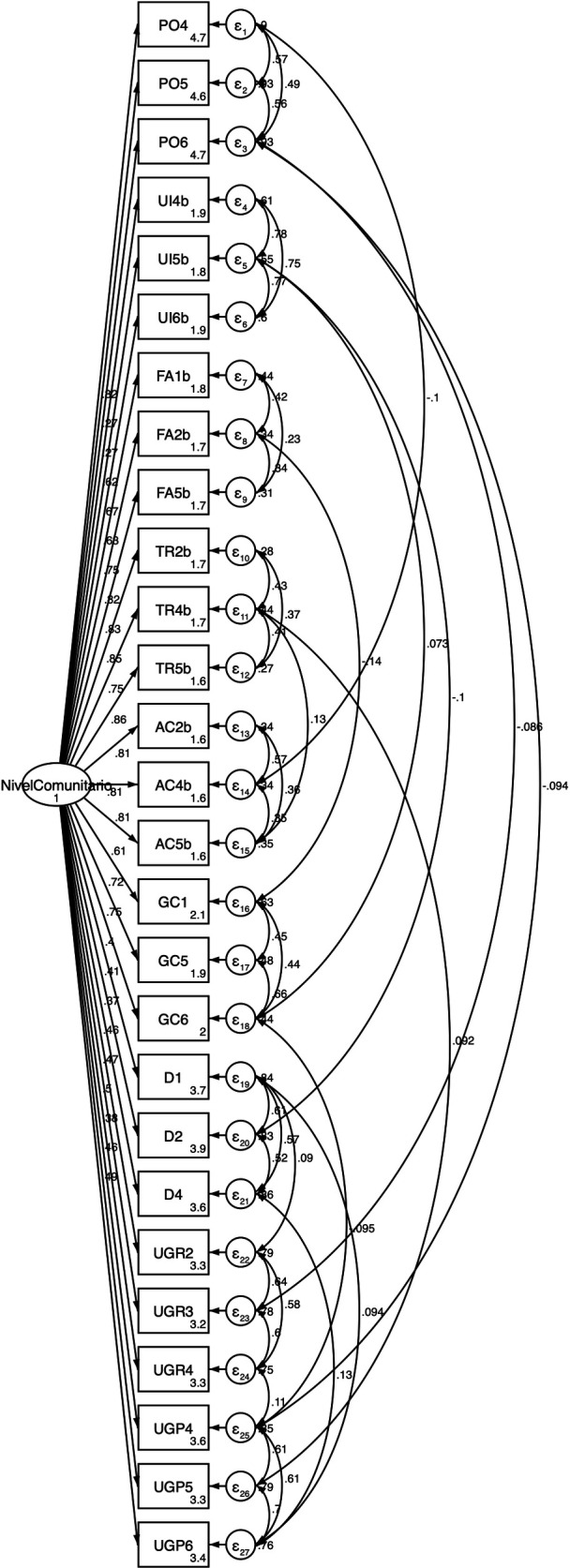
Confirmatory model by structural domain of the Social Capital Related to CKD questionnaire in Mexican Adolescents.

The confirmatory analyses were carried out using structural equation modeling to evaluate the CD and SD model, which confirmed the validity of the CD model (Fig 1. Confirmatory model by cognitive domain of the Social Capital Related to CKD questionnaire in Mexican Adolescents) with a Chi-squared value of 142.99 (p = 0.05, CFI = 0.97, TLI = 0.97, RMSEA = 0.04 and SRMR = 0.077. The confirmatory analysis also validated the SD model (Fig 2. Confirmatory model by structural domain of the Social Capital Related to CKD questionnaire in Mexican Adolescents), with a chi-squared value of 408.29 (p = 0.00, CFI = 0.98, TLI = 0.97, RMSEA = 0.04 and SRMR = 0.07). Moreover, the confirmed models of the SC domains had an adequate goodness-of-fit in all indices (Figs 1 and 2).

The concurrent criterion validation was carried out between items and dimensions, and significant correlations were evident (p ≤ 0.001), with PCCs that ranged from 0.23 to 0.77 for the CD, and from 0.35 to 0.83 for the SD (Table 3). As for the correlation between the SC dimensions and domains, the PCCs ranged from 0.32 to 0.76 for the 6 dimensions of the CD and with PCCs from 0.43 to 0.84 obtained for the SD and its 9 dimensions. All correlations were statistically significant (p ≤ 0.001: Table 4).

**Table 3:** Concurrent validity between items and cognitive and structural domain of the Social Capital Related to CKD questionnaire in Mexican Adolescents.

Items	Cognitive Domain PCC	Items	Structural Domain PCC
GN2	0.6294*	PO4	0.3930*
GN7	0.7734*	PO5	0.3620*
GN8	0.7328*	PO6	0.3521*
SH5	0.5069*	IL4b	0.3521*
SH6	0.4207*	IL5b	0.7140*
SH7	0.5203*	IL6b	0.6859*
SB3	0.4710*	FA1b	0.7339*
SB4	0.5397*	FA2b	0.7857*
SB5	0.5341*	FA5b	0.8127*
PF2	0.3095*	NS2b	0.8183*
PF4	0.4279*	NS4b	0.7456*
PF5	0.4536*	NS5b	0.8310*
S4	0.5666*	CA2b	0.7870*
S5	0.5642*	CA4b	0.7841*
S6	0.4740*	CA5b	0.7908*
T4	0.2606*	DC1	0.6431*
T6	0.2712*	DC5	0.7402*
T7	0.2334*	DC6	0.7643*
		D1	0.5315*
		D2	0.5139*
		D4	0.4869*
		LGR2	0.5540*
		LGR3	0.5704*
		LGR4	0.5983*
		LPG4	0.5071*
		LPG5	0.5707*
		LPG6	0.5936*

PCC: Pearson´s correlation coefficient *p ≤ 0.001.

GN: Generalized Norms, SH: Social Harmony, SB: Sense of Belonging, PF: Perceived Fairness, S: Support, T: Trust, PO: Organizational participation, IL: Institutional Links, FA: Frequency of Actions, NS: Network Size, CA: Collective Action, DC: Degree of Citizenship, D: Diversity, LGR: Links to Groups with Resources, LPG: Links with Parallel Groups.

**Table 4:** Concurrent validity between dimensions and cognitive and structural domain of the Social Capital Related to CKD questionnaire in Mexican Adolescents.

Dimensions	Cognitive Domain PCC	Dimensions	Structural Domain PCC
Generalized Norms	0.7665*	Organizational participation	0.4353*
Social Harmony	0.6007*	Institutional Links	0.7286*
Sense of Belonging	0.5784*	Frequency of Actions	0.8505*
Perceived Fairness	0.5033*	Network Size	0.8571*
Support	0.6222*	Collective Action	0.8448*
Trust	0.3267*	Degree of Citizenship^☼^	0.7888*
		Diversity	0.5865*
		Links to Groups with Resources	0.6401*
		Links with Parallel Groups	0.6209*

PCC: Pearson´s correlation coefficient, *p ≤ 0.001.

GN: Generalized Norms, SH: Social Harmony, SB: Sense of Belonging, PF: Perceived Fairness, S: Support, T: Trust, PO: Organizational participation, IL: Institutional Links, FA: Frequency of Actions, NS: Network Size, CA: Collective Action, DC: Degree of Citizenship, D: Diversity, LGR: Links to Groups with Resources, LPG: Links with Parallel Groups. ☼Degree of Citizenship: This refers to the voluntary capacity of citizens and communities to work together directly, or through elected representatives, to exercise economic, social and political power in the pursuit of shared goals.

With regards reliability, all items showed good internal consistency since they met the criterion for acceptable reliability (αC ≥ 0.70), with αC values ranging from 0.82 to 0.95. An αC = 0.84 was obtained for the CD with 18 items, with αC values for the individual dimensions ranging from 0.70 to 0.84. An αC = 0.95 was obtained for the SD with 27 items, with the αC values for the dimensions ranging from 0.80 to 0.95 (Table 5).

**Table 5:** Reliability of the Social Capital Related to CKD questionnaire in Mexican Adolescents.

Cognitive Domain				Structural Domain			
Items	Cronbach’s Alpha of item	Cronbach’s Alpha by dimension	Cronbach’s Alpha by domain	Items	Cronbach’s Alpha of item	Cronbach’s Alpha by dimension	Cronbach’s Alpha by domain
GN2	0.8277	0.8985	**0.8425**	PO4	0.9493	0.8050	**0.9489**
GN7	0.8182			PO5	0.9495		
GN8	0.8236			PO6	0.9496		
SH5	0.8365	0.7000		IL4b	0.9469	0.9491	
SH6	0.8382			IL5b	0.9465		
SH7	0.8361			IL6b	0.9469		
SB3	0.8343	0.8456		FA1b	0.9462	0.9025	
SB4	0.8326			FA2b	0.9455		
SB5	0.8329			FA5b	0.9451		
PF2	0.8428	0.7000		NS2b	0.9450	0.9231	
PF4	0.8409			NS4b	0.9460		
PF5	0.8365			NS5b	0.9449		
S4	0.8294	0.8263		CA2b	0.9455	0.9407	
S5	0.8305			CA4b	0.9456		
S6	0.8338			CA5b	0.9454		
T4	0.8442	0.7497		DC1	0.9472	0.8962	
T6	0.8402			DC5	0.9461		
T7	0.8414			DC6	0.9458		
				D1	0.9483	0.8402	
				D2	0.9484		
				D4	0.9487		
				LGR2	0.9481	0.8788	
				LGR3	0.9479		
				LGR4	0.9476		
				LPG4	0.9485	0.8810	
				LPG5	0.9479		
				LPG6	0.9477		

GN: Generalized Norms, SH: Social Harmony, SB: Sense of Belonging, PF: Perceived Fairness, S: Support, T: Trust, PO: Organizational participation, IL: Institutional Links, FA: Frequency of Actions, NS: Network Size, CA: Collective Action, DC: Degree of Citizenship, D: Diversity, LGR: Links to Groups with Resources, LPG: Links with Parallel Groups.

## Discussion

A valid and reliable social capital related to CKD questionnaire was developed for Mexican adolescents.

There is no widely accepted definition of SC and on the whole, there is no consensus as to the SC dimensions that should be studied, with the concepts proposed by Bourdieu [67], Coleman [68]and Putnam [1] receiving most attention. SC has mostly been measured by applying the theories of social networks (resources available to individuals through their social connections) and those of social cohesion (the attributes of a social group and the properties of a community, whereby the context influences individuals to cooperate or actively participate [3,69]).

Here, a questionnaire was designed to measure the SC related to CKD in adolescents in a valid and reliable way. This questionnaire is based on Robert Putnam’s concept of SC and on Uphoff’s proposal to measure SC through its CD and SD [2]. The SC is multidimensional and thus, it is necessary to follow theoretical approaches and consider the context of the study (secondary school adolescent students and CKD in this case) in order to measure the SC related to health properly [3]. After an extensive review of the literature, an initial 24 dimensions were identified to measure SC, from which 6 dimensions in the CD and 9 dimensions in the SD were included in the questionnaire given their relevance from a theoretical perspective. The content validation by experts verified the theoretical congruence of each of these items within both dimensions of the questionnaire (57). The degree of agreement among the experts in this study was higher than that reported previously with the content validity index (0.79) [18].

The face validity was assessed through four focus groups, which allowed the content of the items to be simplified and changes to be made based on context as opposed to the theoretical content [50]. By using focus groups, discussions took place and a consensus was reached on the concepts underlying the items that were not clear, which is very important for the questionnaire to be easily understood by the target population. This represents an advantage over other studies that use individual validation techniques [18,51].

According to the results of the KMO test and the Bartlett’s Test of Sphericity, the factor stability was adequate to carry out the PCA [64,65], consistent with earlier data [30]. The communality of all the items in the final models was greater than 0.6 and the factor loadings were greater than 0.40, values considered adequate [70]. The final CD and SD models adequately grouped 3 items in each of their dimensions, indicating a significant proportion of the variance explained by all the items included in the models [71]. These results are superior to those seen previously [30], which may be explained by the fact that all the dimensions in the two domains had a variance close to 10%, a value considered adequate. This is due to the fact that the PCA is more useful when all the variables are measured in the same units and the variances are of similar magnitude [72].

The CFA of the CD and SD models were carried out with structural equation modeling. The values of the CFI, TLI and RMSEA indices for the final models, indicated that their goodness-of-fit was adequate. The value obtained for the SRMR index was also adequate according to Hu and Bentler, who established that a SRMR index value less than 0.09 was optimal [73]. There is only one study that has previously reported this type of analysis, with similar results for the CFI (≥0.90) and RMSEA (≤ 0.05) indices [18].

While the recommended approach in factor analysis is to use independent samples for exploratory (in this case PCA) and confirmatory (CFA) analyses to ensure cross-validation and avoid overfitting [74,75], this could be considered as a possible limitation of the sample size. Some authors acknowledge that the use of the same sample is acceptable provided that the results are interpreted with caution [75–77].

In this study, concurrent criterion validity was determined by assessing the correlation between items within each domain and dimensions of the SC. All correlations were statistically significant (p ≤ 0.001), with PCC values ≥0.5, with the higher the correlation coefficients the better the criterion validity [58]. The items of the trust dimension in the CD and regarding organizational participation in the SD showed weak but statistically significant correlations (PCC = 0.23–0.39; p < 0.001), which may possibly be explained by the items not having a parametric distribution (skewness and kurtosis values > +2 or <−2). This may be due to social desirability being considered as a source of response bias [78], which is seen as a personality trait that uses psychological adjustment to seek compliance with social demands and social approval [79,80]. Moreover, this might predispose the individual to follow social norms in search of harmonious relationships that promote higher self-esteem and a sense of competence [80]. Of the previous studies reviewed, none was found to have assessed this type of validity. Internal consistency was determined through the αC, with adequate values for each of the domains, dimensions and items, indicating that the questionnaire is reliable [60]. Similar results were reported previously in studies on SC [18,23,27,29,30,54,56].

To date, no questionnaire has been designed that is suitable to study the SC that might be related to CKD in adolescent populations and that complies with all the criteria for development, validation and reliability. In an extensive search of the literature, 65 studies were identified that addressed SC in adolescents, 40 of which measured SC in relation to a health-related problem. The data regarding the development, validation and reliability of the questionnaires reported in these studies were analyzed to compare them with those from the present study. In some cases SC questionnaires were developed following the theoretical approach of Putnam [20,22,24,26,30,31,36,39,50] and Kawachi [37], although none of these evaluated the 15 dimensions included in the present study. The dimensions that were included most frequently in these previous studies were: trust [20,26,28,31,35,37–39,43,45,46,49–51], participation [28,31,35,37,43,46,49], generalized norms [20,28,35,38,39,50], support [20,30,46], collective action [51], and sense of belonging [45].

One strength of the measuring instrument developed here is the congruence and solidity throughout the evaluation of its design, validation and reliability in order to ensure better measurement of health related SC. This SC is a complex construct because its multidimensionality depends on the context and the subject under study [3]. When the assessment of exposure or disease status is systematically inaccurate, it can lead to a distortion in the estimate (underestimation or overestimation) of the effect of interest, which is known as information bias [81]. The use of a questionnaire that is valid and reliable could limits the risk of information bias as a result of misclassification.

However, the questionnaire may have some limitations when administered to non-school adolescents whose context may differ, thereby restricting its use. It is therefore recommended that this questionnaire be used essentially for the target population for whom it is designed, and that it should be adapted and validated in future studies to the context of other study populations.

It is important to bear in mind other studies using valid and reliable questionnaires to evaluate SCH in chronic degenerative diseases and in vulnerable populations like adolescents. These studies are important, as described by Kawachi [3–5], since SC is useful to improve or maintain the health of these populations by disseminating relevant information, promoting healthy behaviors, establishing behavioral norms within groups, promoting access to materials and resources, and improving psychosocial processes to provide emotional support yet in a context of mutual respect.

Future research using an approach with a broader vision of CKD will allow the relevant domains and dimensions of SC to be evaluated in adolescents in order to assess the frequency and distribution of these dimensions, the finding of this study should eventually lead to the measuring strength of their association with screening CKD in adolescents.

In conclusion, in the present study a questionnaire was designed and validated and shown to reliably measure the health-related SC through the use of 2 domains and 15 dimensions. Consequently, we propose that this instrument can be used in future studies to evaluate the association of each dimensions of domains the SC with the screening of CKD in adolescents.

## Supporting information

S1 File Questionnaire to assess the health related Social Capital for Chronic Kidney Disease among Mexican adolescents. (PDF)

S2 File Operationalization of Questionnaire to assess the health related Social Capital for Chronic Kidney Disease among Mexican adolescents. (PDF)
