# Supplementary material for: Development and validation of a questionnaire to assess the health related Social Capital for Chronic Kidney Disease among Mexican adolescents
Source: PLoS One. 2025 Jul 21;20(7):e0328386. doi: 10.1371/journal.pone.0328386 (PMC12279121; doi:10.1371/journal.pone.0328386)
Supplement: S1 File — (PDF) [file pone.0328386.s001.pdf]

QUESTIONNAIRE TO ASSESS THE HEALTH-RELATED SOCIAL CAPITAL FOR CHRONIC KIDNEY DISEASE AMONG MEXICAN ADOLESCENTS

1. COGNITIVE DOMAIN

Instructions:

The adolescent will be informed that the following questions are asked based on the assumption that he or she has chronic kidney disease.

1.1.- Generalized Norms

ITEMS	ALWAYS	ALMOST ALWAYS	SOMETIMES	RARELY	NEVER
1.- I talk to my schoolmates or neighbors to help avoid kidney disease and I ask them to also talk about this topic to other schoolmates or neighbors.	5	4	3	2	1
2.- Together with my schoolmates or neighbors, we look to promote groups that act to detect kidney disease and I hope that others are similarly organized.	5	4	3	2	1
3.- I participate in a group of adolescents to avoid complications of kidney disease and I hope that my schoolmates or neighbors also participate in a similar groups.	5	4	3	2	1

1.2.- Social Harmony

ITEMS	FULLY AGREE	AGREE	NEITHER AGREE NOR DISAGREE	DISAGREE	TOTALLY DISAGREE
1.- In my neighborhood, both my neighbors and I can obtain the medications we need to control kidney disease.	5	4	3	2	1
2.- I feel my neighborhood is a supportive community where I can obtain the necessary food to maintain a healthy diet and control kidney disease.	5	4	3	2	1
3.- I feel my neighborhood is a supportive community that makes it easier to obtain the medications I need to treat kidney disease.	5	4	3	2	1

1.3.- Sense of Belonging

ITEMS	FULLY AGREE	AGREE	NEITHER AGREE NOR DISAGREE	DISAGREE	TOTALLY DISAGREE
1.- I would like to be a member of a group that promotes healthy eating in my neighborhood to improve the control of kidney disease.	5	4	3	2	1
2.- I would like to be a member of a group in my neighborhood that, together with the hospitals, promotes compliance with the clinical recommendations for kidney disease to improve disease control.	5	4	3	2	1
3.- I would like to be a member of a group that, together with the hospitals, shares information to improve the control of kidney disease among my schoolmates and neighbors.	5	4	3	2	1

1.4.- Perceived Fairness

ITEMS	FULLY AGREE	AGREE	NEITHER AGREE NOR DISAGREE	DISAGREE	TOTALLY DISAGREE
1.- If my schoolmates or neighbors with kidney disease are allowed to schedule a hospital appointment for Saturdays, I hope that I am allowed to do the same at my hospital.	5	4	3	2	1
2.- If my schoolmates or neighbors with kidney disease wait less than 20 minutes in the hospital's waiting room before being attended by the doctor, I hope to have the same waiting time at my hospital before seeing a doctor.	5	4	3	2	1
3.- If my schoolmates or neighbors have monthly appointments scheduled to monitor their kidney disease, I hope that I also have an appointment with my doctor every month.	5	4	3	2	1

1.5.- Support

ITEMS	FULLY AGREE	AGREE	NEITHER AGREE NOR DISAGREE	DISAGREE	TOTALLY DISAGREE
1.- I consider that I have neighbors who support me by encouraging me to comply with the instructions given to me by the doctor to improve my kidney disease.	5	4	3	2	1
2.- I consider that I have neighbors who will help me schedule a hospital appointment to have my kidney disease monitored.	5	4	3	2	1
3.- I consider that I have neighbors who will help me get my doctor's prescription filled at the hospital's pharmacy to keep my kidney disease controlled.	5	4	3	2	1

1.6.- Trust

ITEMS	TO A GREATEXTENT	QUITE STRONGLY	NEITHER STRONGLY NOR WEAKLY	WEAKLY	NOT AT ALL
1.- I trust the recommendations to improve the management of my kidney disease received from the hospital's healthcare personnel, such as nurses, social workers, nutritionists, etc.	5	4	3	2	1
2.- I trust the information received from the hospitals to avoid kidney disease.	5	4	3	2	1
3.- I trust the information received from private hospital doctors to treat kidney disease.	5	4	3	2	1

2. STRUCTURAL DOMAIN

Instructions:

The adolescent will be informed that the following questions are set out based on the assumption that they form part of a neighborhood group or a committee of classmates with chronic kidney disease.

2.1.- Participation in organizations

ITEMS	FULLY AGREE	AGREE	NEITHER AGREE NOR DISAGREE	DISAGREE	TOTALLY DISAGREE
1.- I think that my schoolmates and neighbors should participate in the development of hospital programs to promote exercise in order to improve the control of kidney disease.	5	4	3	2	1
2.- I think that my neighbors or schoolmates could form groups in the neighborhood to help people follow instructions to manage kidney disease.	5	4	3	2	1
3.- I think that the participation of my schoolmates and neighbors in hospital could reduce the risks associated with kidney disease.	5	4	3	2	1

2.2.- Institutional links

ITEMS	TOTALLY SUFFICIENT	VERY SUFFICIENT	NEITHER TOO FEW NOR TOO MANY	SOMEWHAT INSUFFICIENT	TOTALLY INSUFFICIENT
1.- How would you rate the number of hospitals in which you participate or have participated in activities or programs to promote healthy eating among your classmates and neighbors to improve the control of kidney disease?	5	4	3	2	1
2.- How would you rate the number of hospitals in which you participate or have participated in activities or programs aimed at avoiding complications of kidney disease in your schoolmates and neighbors?	5	4	3	2	1
3.- How would you rate the number of hospitals in which you participate or have participated in activities or programs offering support to comply with the treatments to improve kidney disease control among your classmates and neighbors?	5	4	3	2	1

2.3.- Frequency of action

ITEMS	TOTALLY SUFFICIENT	VERY SUFFICIENT	NEITHER TOO FEW NOR TOO MANY	SOMEWHAT INSUFFICIENT	TOTALLY INSUFFICIENT
1.- How would you rate the number of times during the last 7 days that you have talked to your schoolmates or neighbors to encourage compliance with treatment for kidney disease?	5	4	3	2	1
2.- How would you rate the number of times during the last 7 days that you have talked to your schoolmates or neighbors about the information you have about the diagnosis of kidney disease?	5	4	3	2	1
3.- How would you rate the number times during the last 7 days that you have met with your schoolmates or neighbors to talk about how to promote healthy eating for people with kidney disease?	5	4	3	2	1

2.4.- Network size

ITEMS	TOTALLY SUFFICIENT	VERY SUFFICIENT	NEITHER TOO FEW NOR TOO MANY	SOMEWHAT INSUFFICIENT	TOTALLY INSUFFICIENT
1.- How would you rate the number of schoolmates or neighbors with whom you have contacted during the last 7 days to participate in activities aimed at promoting the healthy eating recommended for people with kidney disease?	5	4	3	2	1
2.- How would you rate the number of schoolmates or neighbors with whom you have contacted during the last 7 days to participate in activities aimed at promoting the exercise recommended for people kidney disease?	5	4	3	2	1
3.- How would you rate the number of schoolmates or neighbors with whom you have contacted during the last 7 days to participate in activities aimed at encouraging compliance with the treatments of kidney disease?	5	4	3	2	1

2.5.- Collective Action

ITEMS	TOTALLY SUFFICIENT	VERY SUFFICIENT	NEITHER TOO FEW NOR TOO MANY	SOMEWHAT INSUFFICIENT	TOTALLY INSUFFICIENT
1.- How would you rate the number of times that you have met with schoolmates or neighbors in the past 3 months to go to a hospital to get information about kidney disease?	5	4	3	2	1
2.- How would you rate the number of times that you, together with your schoolmates or neighbors, have gone to hospital talks promoting activities to control kidney disease?	5	4	3	2	1
3.- How would you rate the number of times that hospitals have given talks in your neighborhood to promote healthy eating for kidney disease?	5	4	3	2	1

2.6.- Degree of Citizenship

ITEMS	ALWAYS	ALMOST ALWAYS	SOMETIMES	RARELY	NEVER
1.- How often do you and your schoolmates or neighbors collaborate with the school's Principal and teachers, or neighborhood leaders, to negotiate programs that promote healthy eating for people with kidney disease?	5	4	3	2	1
2.- How often do you and your schoolmates or neighbors collaborate with the school's Principal and teachers, or neighborhood leaders, to negotiate programs that encourage compliance with the treatments for kidney disease in others schoolmates or neighbors?	5	4	3	2	1
3.- How often do you and your schoolmates or neighbors collaborate with the school's Principal and teachers, or neighborhood leaders, to negotiate programs that support and inform peoples as to how they can improve their kidney disease in others schoolmates or neighbors?	5	4	3	2	1

2.7.- Diversity

ITEMS	FULLY AGREE	AGREE	NEITHER AGREE NOR DISAGREE	DISAGREE	TOTALLY DISAGREE
1.- I, along with my schoolmates or neighbors, would agree to share information with other groups of adolescents about the activities that have helped us better control kidney disease.	5	4	3	2	1
2.- I, along with my schoolmates or neighbors, would agree to share information with other groups of adolescents about the activities that have helped us promote healthy eating to control kidney disease.	5	4	3	2	1
3.- I, along with my schoolmates or neighbors, would agree to share information with other groups of adolescents about the activities that have helped us comply with the medical treatment to better control kidney disease.	5	4	3	2	1

2.8.- Links to groups with resources

ITEMS	FULLY AGREE	AGREE	NEITHER AGREE NOR DISAGREE	DISAGREE	TOTALLY DISAGREE
1.- Do you feel part of your group at school or in your neighborhood as to motivate you and encourage compliance with the treatment of kidney disease?	5	4	3	2	1
2.- Do you feel part of your group at school or in your neighborhood as to motivate you and negotiate with your schoolmates or neighbors to get support for programs aimed to prevent kidney disease?	5	4	3	2	1
3.- Do you feel part of your group at school or in your neighborhood as to motivate you and promote healthy eating with your schoolmates or neighbors with kidney disease?	5	4	3	2	1

2.9.- Links to parallel groups

ITEMS	FULLY AGREE	AGREE	NEITHER AGREE NOR DISAGREE	DISAGREE	TOTALLY DISAGREE
1.- Do you think that your group at school or in your neighborhood are part of the hospital, civil associations or support groups aimed at promoting exercise as a means to improve kidney disease?	5	4	3	2	1
2.- Do you think that your group at school or in your neighborhood are part of the hospital, civil associations or support groups that promote healthy eating to improve kidney disease?	5	4	3	2	1
3.- Do you think that your group at school or in your neighborhood are part of the hospital, civil associations or support groups to pursue better medical care for people with kidney disease?	5	4	3	2	1
