# Supplementary material for: Development and validation of a questionnaire to assess the health related Social Capital for Chronic Kidney Disease among Mexican adolescents
Source: PLoS One. 2025 Jul 21;20(7):e0328386. doi: 10.1371/journal.pone.0328386 (PMC12279121; doi:10.1371/journal.pone.0328386)
Supplement: S2 File — (PDF) [file pone.0328386.s002.pdf]

OPERATIONALIZATION OF QUESTIONNAIRE TO ASSESS THE HEALTH-RELATED SOCIAL CAPITAL FOR CHRONIC KIDNEY DISEASE AMONG MEXICAN ADOLESCENTS

Concept	Domains	Dimensions	Variability	Indicators	Scale	Modification of items by experts
Social Capital and Health:<br>The features of social organization, like networks, norms and social trust, that facilitate coordination and cooperation for mutual benefit in relation to the presence and risks of Chronic Kidney Disease [1].	**TYPES OF SOCIAL CAPITAL**<br>**COGNITIVE DOMAIN:**<br>Derived from mental processes and the resulting ideas, reinforced by culture and ideology, specifically norms, values, attitudes and beliefs that contribute to cooperative behavior, including the control of risk behavior, provision of mutual aid and support, and informal means for the exchange of information [2].	**A.1.- Generalized Norms:** Norms of reciprocity among citizens in a community that determine the extent of cooperative and mutually beneficial behaviors within the community [3].	The extent of cooperative and mutually benefit. Reciprocal actions undertaken between school classmates and neighbors in their community [3].	This parameter is measured through the reciprocity of cooperative well-being among individuals [3].	5-point Likert scale based on the adolescent's behavior:<br>Always,<br>Almost always,<br>Sometimes,<br>Rarely,<br>Never.	1.- I talk to my schoolmates or neighbors to help avoid kidney disease and I ask them to also talk about this topic to other schoolmates or neighbors.<br>2.- Together with my schoolmates or neighbors, we look to promote groups that act to detect kidney disease and I hope that others are similarly organized.<br>3.- I participate in a group of adolescents to avoid complications of kidney disease and I hope that my schoolmates or neighbors also participate in a similar groups.
		**A.2.- Social harmony:** Feeling at home in the neighborhood, perceiving the community as a place to live, attachment to the neighborhood, integration within the community [4].	Perceiving the community as a good place to live [4]. It reflects the adolescent's sensation that this is true of their school and community.	The perception adolescents have of their community and of whether it is a good place to live [4].	5-point Likert scale based on the attitudes, feelings and/or perceptions:<br>Fully agree,<br>Agree,<br>Neither agree nor disagree,<br>Disagree,<br>Totally disagree.	1.- In my neighborhood, both my neighbors and I can obtain the medications we need to control kidney disease.<br>2.- I feel my neighborhood is a supportive community where I can obtain the necessary food to maintain a healthy diet and control kidney disease.<br>3.- I feel my neighborhood is a supportive community that makes it easier to obtain the medications I need to treat kidney disease.
		**A.3.- Sense of Belonging:** Attitudes related to participation in voluntary organizations, involvement in social clubs or political groups and civic engagement, providing help to others [5].	Attitudes that favor or that go against participation in voluntary organizations, social clubs or groups that seek to promote health in the community [5].	Emotions and beliefs that elicit favorable or unfavorable reactions when belonging to an organization or social group that promotes the health of adolescents [5].	5-point Likert scale based on attitudes, feelings and/or perceptions:<br>Fully agree,<br>Agree,<br>Neither agree nor disagree,<br>Disagree,<br>Totally disagree.	1.- I would like to be a member of a group that promotes healthy eating in my neighborhood to improve the control of kidney disease.<br>2.- I would like to be a member of a group in my neighborhood that, together with the hospitals, promotes compliance with the clinical recommendations for kidney disease to improve disease control.<br>3.- I would like to be a member of a group that, together with the hospitals, shares information to improve the control of kidney disease among my schoolmates and neighbors.
		**A.4.- Perceived Fairness:** Equal opportunities, equality and dignity without discrimination [6]. Responsibility for the actions of decision makers in both the public and private sectors to pursue policies and	Degree of Equity - Equity in healthcare:<br>When equal access to healthcare exists for the same healthcare needs, achieving equivalent use of healthcare services for the same health need and	Equity in access to healthcare:<br>Equal access to healthcare for the same health needs of adolescents (Coverage, organizational, and system/demand barriers).	5-point Likert scale based on attitudes, feelings and/or perceptions:<br>Fully agree,<br>Agree,<br>Neither agree nor disagree,	1.- If my schoolmates or neighbors with kidney disease are allowed to schedule a hospital appointment for Saturdays, I hope that I am allowed to do the same at my hospital.<br>2.- If my schoolmates or neighbors with kidney disease wait less than 20 minutes in the hospital's waiting room before being attended by the doctor, I hope to have the same waiting time at my hospital before seeing a doctor.

		practices that promote and protect health.	equivalent quality of healthcare offered for the same health need [7].	Equal use of healthcare services for the same health need (Frequency of use).<br>Equivalent quality of healthcare services offered for the same healthcare need (Technical aspects of quality of care).<br>Equal access to healthcare services, equivalent use of healthcare services and equivalent quality of healthcare services.	Disagree,<br>Totally disagree.	3.- If my schoolmates or neighbors have monthly appointments scheduled to monitor their kidney disease, I hope that I also have an appointment with my doctor every month.
		**A.5.- Support:**<br>It is the interaction between people to receive and give help (social, emotional, instrumental and informational)[8].	✓ Social support:<br>Existence or availability of people who can be trusted and people who let us know we are important [9].<br>✓ Emotional support: Expressions of empathy, love and trust [10].<br>✓ Instrumental support:<br>Behaviors directly aimed at solving the adolescent's problem [11]<br>✓ Informational support:<br>Receiving useful information to face the problem [11].	* Social support:<br>This is the perception that there are sufficient of neighbors or schoolmates available whom one can turn to in times of need [9].<br>*Emotional support:<br>Identification of feelings (empathy) in the adolescents that helps them maintain friendships [10].<br>*Instrumental support:<br>Identification of behaviors aimed at solving adolescent problems [11].<br>*Informational support:<br>Reflects the useful information received to solve the problem [11].	5-point Likert scale based on attitudes, feelings and/or perceptions:<br>Fully agree,<br>Agree,<br>Neither agree nor disagree,<br>Disagree,<br>Totally disagree.	1.- I consider that I have neighbors who support me by encouraging me to comply with the instructions given to me by the doctor to improve my kidney disease.<br>2.- I consider that I have neighbors who will help me schedule a hospital appointment to have my kidney disease monitored.<br>3.- I consider that I have neighbors who will help me get my doctor's prescription filled at the hospital's pharmacy to keep my kidney disease controlled.
		**A.6.- Trust:**<br>Attitude based on the behavior expected from a person (adolescent) who is participating in a relationship with healthcare personnel. It is reinforced by the cultural principle of reciprocity [12].	Reciprocity as the fundamental principle formal and informal institutional relationships at the level of trust [12].	The extent to which adolescent's trust people, institutions and organizations related to chronic kidney disease [12].	5-point Likert scale based on perceptions:<br>To a greatextent,<br>Quite strongly,<br>Neither strongly nor weakly,<br>Weakly,<br>Not at all.	1.- I trust the recommendations to improve the management of my kidney disease received from the hospital's healthcare personnel, such as nurses, social workers, nutritionists, etc.<br>2.- I trust the information received from the hospitals to avoid kidney disease.<br>3.- I trust the information received from private hospital doctors to treat kidney disease.
	**STRUCTURAL DOMAIN:**<br>The structural components of social capital include the roles, rules, precedents and procedures, as well as a	**B.1.- Participation in organizations:**<br>Organizations and networks through which citizens access collective decision-making	Participation in decision-making processes of organizations and networks [4].	The participation of adolescents in decision-making within organizations and support networks that	5-point Likert scale based on attitudes, feelings and/or perceptions:<br>Fully agree,	1.- I think that my schoolmates and neighbors should participate in the development of hospital programs to promote exercise in order to improve the control of kidney disease.<br>2.- I think that my neighbors or schoolmates could form groups in the neighborhood to help people follow instructions to manage kidney disease.

	wide variety of networks that contribute to cooperation. At the macro level, it is determined by general health policies, the mechanisms for their implementation and the institutional agents that implement them. At the meso level, it is determined by factors of diverse networks involved in the delivery and dissemination of the services to obtain health-related information [2].	processes that are translated into local social policies and programs, intensifying social relationships, while improving trust and expectations of mutual benefit [4].		aim to improve aspects of chronic kidney disease [4].	Agree,<br>Neither agree nor disagree,<br>Disagree,<br>Totally disagree.	3.- I think that the participation of my schoolmates and neighbors in hospital could reduce the risks associated with kidney disease.
		**B.2.- Institutional links:** Links between people and authorities [13].	The capacity of individuals to forge bonds with those who come from a higher or lower social status [13].	Whether adolescents are able to establish collaborative links with healthcare institutions that promote healthy lifestyles for chronic kidney disease (the number of institutions with which they are linked) ([13].	Discrete scale:<br>The number of institutions with which the adolescent is linked<br><br>5-point Likert scale based on the perception of the number of institutions with which the adolescent is linked:<br>Totally Sufficient<br>Very Sufficient<br>Neither Too Few Nor Too Many<br>Somewhat Insufficient<br>Totally Insufficient	1.- How would you rate the number of hospitals in which you participate or have participated in activities or programs to promote healthy eating among your classmates and neighbors to improve the control of kidney disease?<br>2.- How would you rate the number of hospitals in which you participate or have participated in activities or programs aimed at avoiding complications of kidney disease in your schoolmates and neighbors?<br>3.- How would you rate the number of hospitals in which you participate or have participated in activities or programs offering support to comply with the treatments to improve kidney disease control among your classmates and neighbors?
		**B.3.- Frequency of action:** Number of times an individual interacts with other contacts and the quality of his/her relationship with people within his/her immediate circle [14].	Number of times an individual interacts with other contacts and the quality of their relationships with people within their immediate circle [14].	The number of times the adolescent interacts with other classmates or neighbors, and the quality of those relationships in their neighborhood, and how they rate the number of times they meet their neighbors [14].	Discrete scale:<br>The number of informal ties<br><br>5-point Likert scale based on the perception of the number of informal ties<br>Totally Sufficient<br>Very Sufficient	1.- How would you rate the number of times during the last 7 days that you have talked to your schoolmates or neighbors to encourage compliance with treatment for kidney disease?<br>2.- How would you rate the number of times during the last 7 days that you have talked to your schoolmates or neighbors about the information you have about the diagnosis of kidney disease?<br>3.- How would you rate the number times during the last 7 days that you have met with your schoolmates or neighbors to talk about how to promote healthy eating for people with kidney disease?

					Neither Too Few Nor Too Many<br>Somewhat Insufficient<br>Totally Insufficient.	
		**B.4.- Network size:**<br>The number of people with whom social contact is maintained, including those who are only there when they are needed [15].	Measurement of network size, density and diversity [15].	The number of schoolmates or neighbors with whom the adolescent has maintained social contact during the last 7 days aimed to improve their schoolmates' chronic kidney disease, including with those schoolmates or neighbors who are only there when they are needed. How they rate the number of the aforementioned schoolmates or neighbors contacted [15].	Discrete scale:<br>The number of members in the group<br><br>5-point Likert scale based on the perception of the number of members in the group:<br>Totally Sufficient<br>Very Sufficient<br>Neither Too Few Nor Too Many<br>Somewhat Insufficient<br>Totally Insufficient.	1.- How would you rate the number of schoolmates or neighbors with whom you have contacted during the last 7 days to participate in activities aimed at promoting the healthy eating recommended for people with kidney disease?<br>2.- How would you rate the number of schoolmates or neighbors with whom you have contacted during the last 7 days to participate in activities aimed at promoting the exercise recommended for people kidney disease?<br>3.- How would you rate the number of schoolmates or neighbors with whom you have contacted during the last 7 days to participate in activities aimed at encouraging compliance with the treatments of kidney disease?
		**B.5.- Collective Action:** This refers to the tendency of a community to cooperate in pursuit of the common good [16].	Norms of mutual cooperation. Measures of such norms that are the result of customs and traditions *[16].*	The efforts and cooperation among adolescents that seek to improve their schoolmates or neighbors affected by chronic kidney disease, and how they rate the number of times they have met with them [16].	Discrete scale:<br>The number of times<br><br>5-point Likert scale based on the perception of the number of times<br>Totally Sufficient<br>Very Sufficient<br>Neither Too Few Nor Too Many<br>Somewhat Insufficient<br>Totally Insufficient	1.- How would you rate the number of times that you have met with schoolmates or neighbors in the past 3 months to go to a hospital to get information about kidney disease?<br>2.- How would you rate the number of times that you, together with your schoolmates or neighbors, have gone to hospital talks promoting activities to control kidney disease?<br>3.- How would you rate the number of times that hospitals have given talks in your neighborhood to promote healthy eating for kidney disease?
		**B.6.- Degree of Citizenship:**<br>This refers to the voluntary capacity of citizens and communities to work together directly, or through elected representatives, in order to exercise economic,	Voluntary capacity to work together directly or through representatives to achieve a common goal [17].	The capacity of adolescents to work together with school authorities and community leaders in order to carry out activities that help improve their schoolmates or neighbors' chronic kidney disease [17].	5-point Likert scale based on the adolescent's behavior:<br>Always,<br>Almost always,<br>Sometimes,<br>Rarely,	1.- How often do you and your schoolmates or neighbors collaborate with the school's Principal and teachers, or neighborhood leaders, to negotiate programs that promote healthy eating for people with kidney disease?<br>2.- How often do you and your schoolmates or neighbors collaborate with the school's Principal and teachers, or neighborhood leaders, to negotiate programs that encourage compliance with the treatments for kidney disease in others schoolmates or neighbors?<br>3.- How often do you and your schoolmates or neighbors collaborate with the school's Principal and teachers, or neighborhood leaders, to negotiate

		social and political power in the pursuit of shared goals [17].			Never.	programs that support and inform peoples as to how they can improve their kidney disease in others schoolmates or neighbors?
		**B.7.- Diversity:** The potential to generate ties, social trust and subjective well-being in the different groups of networks [18].	Variety of networks and the ties established in different groups [18].	The diversity of the ties established between different groups of adolescents, schoolmates or neighbors, with a view to improve their chronic kidney disease [18].	5-point Likert scale based on attitudes, feelings and/or perceptions: Fully agree, Agree, Neither agree nor disagree, Disagree, Totally disagree	1.- I, along with my schoolmates or neighbors, would agree to share information with other groups of adolescents about the activities that have helped us better control kidney disease. 2.- I, along with my schoolmates or neighbors, would agree to share information with other groups of adolescents about the activities that have helped us promote healthy eating to control kidney disease. 3.- I, along with my schoolmates or neighbours, would agree to share information with other groups of adolescents about the activities that have helped us comply with the medical treatment to better control kidney disease.
		**B.8.- Links to groups with resources:** The extent to which a neighbor integrates into a network (or various networks) within the neighborhood [19]	The individual's degree of network integration [19].	Perception of the extent to which one feels integrated (degree of integration into networks) into a group of schoolmates or neighborhood committee to improve chronic kidney disease [19].	-point Likert scale based on attitudes, feelings and/or perceptions: Fully agree, Agree, Neither agree nor disagree, Disagree, Totally disagree.	1.- Do you feel part of your group at school or in your neighborhood as to motivate you and encourage compliance with the treatment of kidney disease? 2.- Do you feel part of your group at school or in your neighborhood as to motivate you and negotiate with your schoolmates or neighbors to get support for programs aimed to prevent kidney disease? 3.- Do you feel part of your group at school or in your neighborhood as to motivate you and promote healthy eating with your schoolmates or neighbors with kidney disease?
		**B.9.- Links to parallel groups:** This refers to the full range of institutions found in society at large. The higher the degree of institutional completeness within a community, in terms of businesses, churches, banks and social services, the more it offers the newcomer and established member to strengthen links and ties [20].	Institutional links in reference to the extent to which people maintain relationships with a variety of institutions (e.g., with the government bodies, corporations, etc.: [20].	Perception of the extent to which your schoolmates or your neighborhood health committee are integrated into healthcare institutions so that together, they can help improve chronic kidney disease [20].	5-point Likert scale based on attitudes, feelings and/or perceptions: Fully agree, Agree, Neither agree nor disagree, Disagree, Totally disagree.	1.- Do you think that your group at school or in your neighborhood are part of the hospital, civil associations or support groups aimed at promoting exercise as a means to improve kidney disease? 2.- Do you think that your group at school or in your neighborhood are part of the hospital, civil associations or support groups that promote healthy eating to improve kidney disease? 3.- Do you think that your group at school or in your neighborhood are part of the hospital, civil associations or support groups to pursue better medical care for people with kidney disease?

REFERENCES

1. Putnam RD, Leonardi R, Nanetti RY. Making democracy work: Civic traditions in modern Italy: Princeton university press; 1994.
2. Uphoff N. Understanding social capital: learning from the analysis and experience of participation. Social capital: A multifaceted perspective. 2000:215-49.
3. Hendryx MS, Ahern MM, Lovrich NP, McCurdy AH. Access to health care and community social capital. HEALTH SERVICES RESEARCH-CHICAGO-. 2002;37(1):87-104.
4. De Silva M. Context and composition? Social capital and maternal mental health in low income countries: London School of Hygiene and Tropical Medicine; 2005.
5. Bryant PC-a, Norris D. Measurement of social capital: The Canadian experience. 2002.

6. Melton GB. Mental health and social justice: A vision for the 21st century. American journal of orthopsychiatry. 2003;73(3):245-7.
7. Whitehead M. The concepts and principles of equity and health. International journal of health services. 1992;22(3):429-45.
8. Aranda C, Pando M. Conceptualization of the social support and the social support network. 2013.
9. Orviz S, Novoa T, Palacios M. Evaluación de las Habilidades Sociales en Rehabilitación Psicosocial. Evaluación en Rehabilitación Psicosocial Valladolid: FEARP. 2010.
10. Uchida Y, Kitayama S, Mesquita B, Reyes JAS, Morling B. Is perceived emotional support beneficial? Well-being and health in independent and interdependent cultures. Personality and Social Psychology Bulletin. 2008;34(6):741-54.
11. Smith MS, Wallston K. How to measure the value of health. Health Education Research. 1992;7(1):129-35.
12. Durston J. Capital social: definiciones, controversias, tipologías. El capital social campesino en la gestión del desarrollo rural Díadas, equipos, puentes y escaleras Libros de la CEPAL. 2002;(69):15-42.
13. Williams E. Measuring religious social capital: The scale properties of the Williams Religious Social Capital Index (WRSCI) among cathedral congregations. Journal of Beliefs & Values. 2008;29(3):327-32.
14. Kemenade S. Social capital as a health determinant: How is it measured. Health Canada, Working Paper. 2003:02-8.
15. Stone W, Hughes J, editors. Measuring Social capital: Toward a standardised approach. Paper presented at the (Vol 3, No 9214, p 7820); 2002.
16. Gertler P, Levine DI, Moretti E. Is social capital the capital of the poor? The role of family and community in helping insure living standards against health shocks. CESifo Economic Studies. 2006;52(3):455-99.
17. Hoskins BL, Mascherini M. Measuring active citizenship through the development of a composite indicator. Social Indicators Research. 2009;90(3):459-88.
18. Vanhoutte B, Hooghe M. Integration in Social Networks as a form of Social Capital: Evidence from a Belgian survey on Social cohesion. status: published. 2009.
19. Sarhimaa A. Social Network Theory as a framework for studying minor Finnic languages with special reference to Karelian. The Quasquicentennial of the Finno-Ugrian Society Helsinki. 2009:161-90.
20. Galabuzi G-E, Teelucksingh C. Social cohesion, social exclusion, social capital: Region of Peel, Human Services; 2010.
